# Supplementary material for: Functional Comparison of Blood-Derived Human Neural Progenitor Cells
Source: Int J Mol Sci. 2020 Nov 30;21(23):9118. doi: 10.3390/ijms21239118 (PMC7730078; doi:10.3390/ijms21239118)
Supplement: Supplementary file 1 [file ijms-21-09118-s001.pdf]

International Journal of
Molecular Sciences

Supplementary Materials

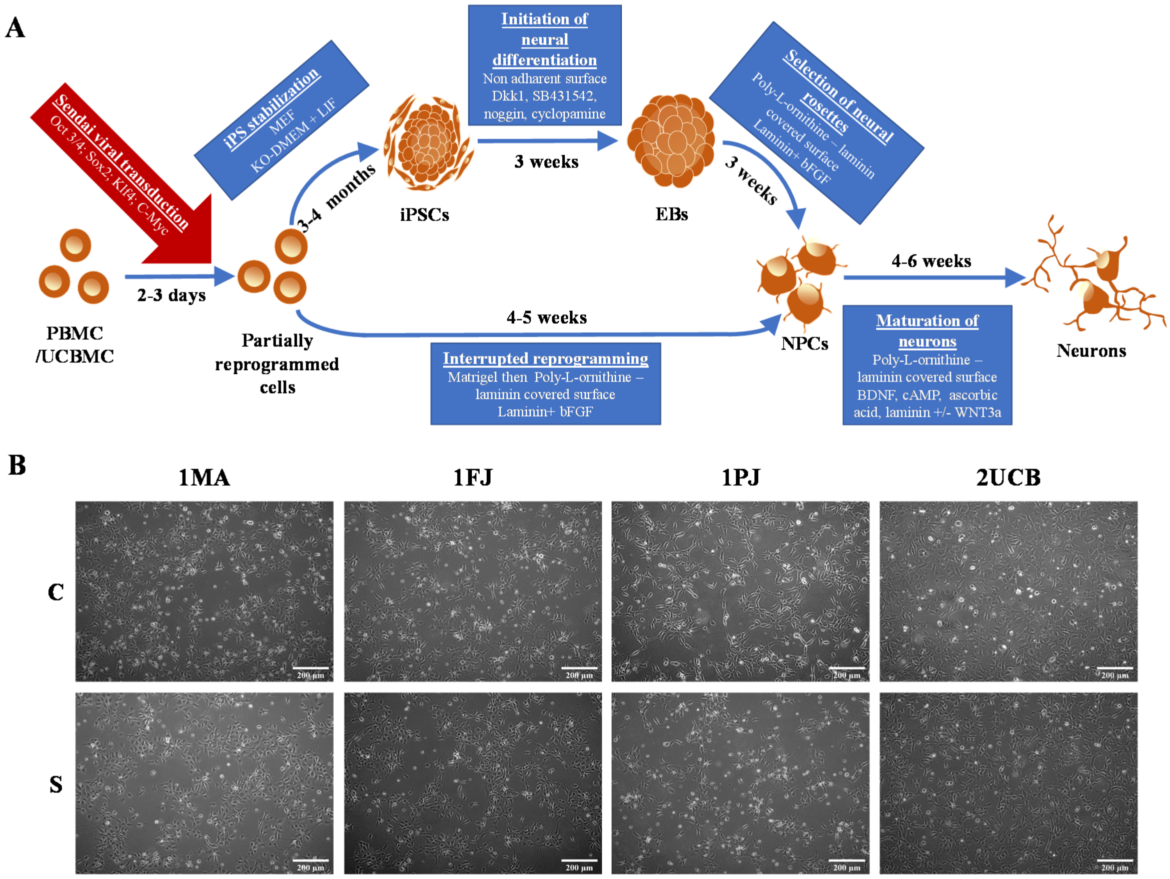

Figure S1. Schematic presentation of NPC differentiation methods. (**A**) The workflow of the differentiation protocols with time line and required materials and the clinical data of donors are presented. (**B**) Representative images of NPCs' morphology. (Scale bar 200 µm).

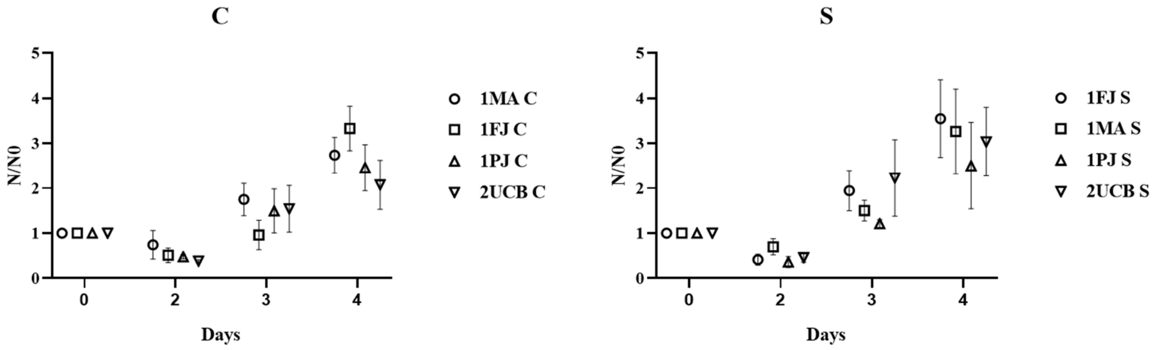

Figure S2. Comparison of the proliferative capacity of progenitors by 3 parallel FCM measurements. Cell counts relative to seeded cell numbers± SEM are plotted per day; all cNPC-s and sNPCs derived are stacked side by side.

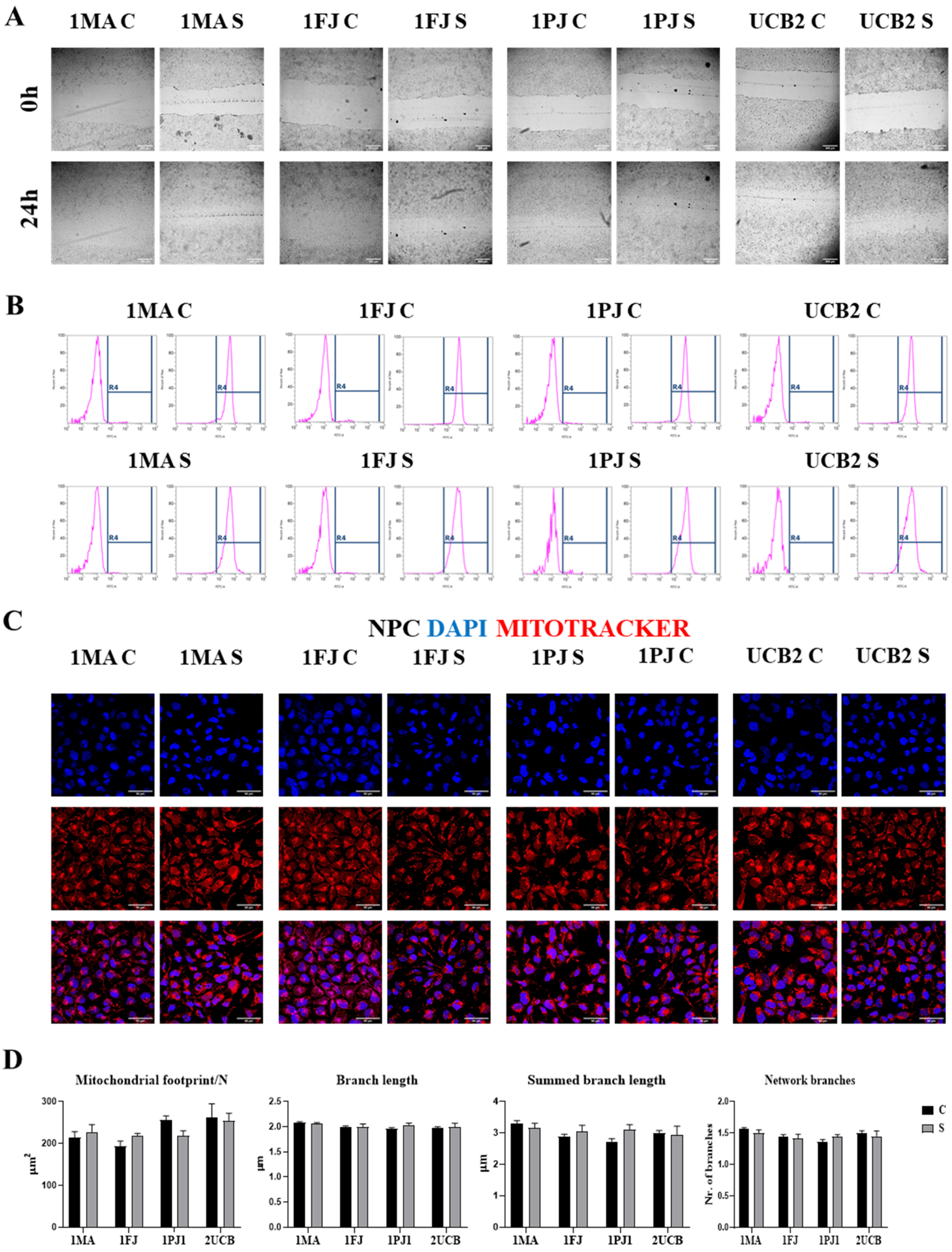

Figure S3. Characterization of NPCs by functional assays. (**A**) Scratch assay: Representative images taken after scratching and 24 hours later. (**B**) Levels of ROS accumulation compared by CELLROX™ green reagent. Representative histograms of green fluorescence intensity are shown on NPC samples without and after incubation with CELLROX™ reagent. (**C**) Mitochondrial functions compared by MitoTracker™ red reagent. Representative confocal images of NPCs cultures - priory plated on 8 well confocal chambers - incubated with the reagent (red), subsequently fixed by PFA, nuclei counterstained by DAPI (blue). (**D**) Morphological analysis of mitochondria in cNPCs and sNPCs calculated by Mitochondrial NetworkAnalysis (MiNA) toolset of ImageJ.

Supplementary Tables

Table S1. Statistical analysis of RT-PCR data.

Donor	Turkey's Multiple Comparison	Nanog	Sox2	Nestin	Pax6	FoxG1
1MA	iPSC vs. NPC C	0.0065	0.0932	0.0115 *	0.0036	0.7353
	iPSC vs. NPC S	0.0039	0.0653	0.0024	0.0034	0.0468
	NPC C vs. NPC S	0.3532	0.8727	0.0278	0.9770	0.0782
1FJ	iPSC vs. NPC C	0.0009	0.0005	0.0186	0.2205	0.0055
	iPSC vs. NPC S	0.0009	0.1469	0.0236	0.0006	0.0053
	NPC C vs. NPC S	0.8993	0.0007	0.8755	0.0009	0.9894
1PJ	iPSC vs. NPC C	0.0061	0.0079	0.0003	0.0341	0.0049
	iPSC vs. NPC S	0.0051	0.0076	0.0028	0.0005	<0.0001
	NPC C vs. NPC S	0.8465	0.9929	0.0020	0.0009	0.0001
UCB2	iPSC vs. NPC C	0.0009	0.0515	0.0052	0.0004	0.0303
	iPSC vs. NPC S	0.0009	0.0640	0.0107	0.0335	0.0010
	NPC C vs. NPC S	0.8993	0.9404	0.2320	0.0009	0.0028

Table S2. Statistical analysis of proliferation.

Source of Variation	*p* Value Day 2	*p* Value Day 3	*p* Value Day 4
Interaction	0.9254	0.4285	0.9233
Donor (PJ,FA,MA,UCB2)	0.2162	0.6807	0.5283
Differentiation (shortcut, classical)	0.6846	0.3979	0.4003

Table S3. Statistical analysis of mitochondrial morphology.

Source of Variation	Mitochondrial Footprint/n	Mean Branch Length	Summed Branch Length	Network Branches
Interaction	0.3812	0.5751	0.2521	0.4036
Donor (PJ, FA, MA, UCB2)	0.0622	0.1521	0.1242	0.0630
Differentiation (shortcut, classical)	0.9067	0.4902	0.4084	0.7055

Table S4. Statistical analysis of Ca-signals evoked by Glutamate.

Tukey's Multiple Comparison	GLU Signal Intensity for 50 Frames	GLU Signal Intensity Max
1MA:C vs. 1MA:S	>0.9999	0.9997
1MA:C vs. 1FJ:C	>0.9999	>0.9999
1MA:C vs. 1FJ:S	0.9838	0.9976
1MA:C vs. 1PJ:C	0.9993	0.9883
1MA:C vs. 1PJ:S	0.9997	0.9952
1MA:C vs. 2UCB:C	0.0236	0.086
1MA:C vs. 2UCB:S	0.9857	0.9972
1MA:S vs. 1FJ:C	0.9998	0.996
1MA:S vs. 1FJ:S	0.9938	0.9598
1MA:S vs. 1PJ:C	>0.9999	0.8975
1MA:S vs. 1PJ:S	>0.9999	>0.9999
1MA:S vs. 2UCB:C	0.0164	0.2043
1MA:S vs. 2UCB:S	0.967	>0.9999
1FJ:C vs. 1FJ:S	0.9554	>0.9999
1FJ:C vs. 1PJ:C	0.9937	0.9997
1FJ:C vs. 1PJ:S	0.9962	0.9784
1FJ:C vs. 2UCB:C	0.1064	0.0889
1FJ:C vs. 2UCB:S	0.9996	0.9851
1FJ:S vs. 1PJ:C	>0.9999	>0.9999
1FJ:S vs. 1PJ:S	0.9996	0.8915
1FJ:S vs. 2UCB:C	0.0063	0.0416
1FJ:S vs. 2UCB:S	0.7207	0.9198
1PJ:C vs. 1PJ:S	>0.9999	0.7816
1PJ:C vs. 2UCB:C	0.0109	0.0196
1PJ:C vs. 2UCB:S	0.878	0.8354
1PJ:S vs. 2UCB:C	0.0076	0.3123
1PJ:S vs. 2UCB:S	0.8864	>0.9999
2UCB:C vs. 2UCB:S	0.2302	0.3842
